# FUT2 inhibits the EMT and metastasis of colorectal cancer by increasing LRP1 fucosylation

**DOI:** 10.1186/s12964-023-01060-0

**Published:** 2023-03-27

**Authors:** Lingnan He, Zijun Guo, Weijun Wang, Shuxin Tian, Rong Lin

**Affiliations:** 1 Department of Gastroenterology, Union Hospital, Tongji Medical College, Huazhong University of Science and Technology, Wuhan, 430022 China; 2 Endoscopy Center, Department of Gastroenterology, Shanghai East Hospital, Tongji University School of Medicine, 150 Jimo Road, Pudong New Area, Shanghai, China; 3 Department of Gastroenterology, Zhongnan Hospital of Wuhan University, Wuhan, China

**Keywords:** Fucosyltransferase 2(FUT2), Fucosylation, Metastasis, Low-density lipoprotein receptor-related protein-1(LRP1), Epithelial-to-mesenchymal transition (EMT)

## Abstract

**Background:**

Fucosyltransferase 2(FUT2) and its induced α-1,2 fucosylation is associated with cancer metastasis. However, the role of FUT2 in colorectal cancer (CRC) metastasis remains unclear.

**Methods:**

The expression levels and clinical analyses of FUT2 were assessed in CRC samples. Migration and invasion assays, EMT detection, nude mice peritoneal dissemination models and intestinal specific FUT2 knockout mice (FUT2^△IEC^ mice) were used to investigate the effect of FUT2 on metastasis in colorectal cancer. Quantitative proteomics study of glycosylated protein, UEA enrichment, Co-immunoprecipitation identified the mediator of the invasive-inhibiting effects of FUT2.

**Results:**

FUT2 is downregulated in CRC tissues and is positively correlated with the survival of CRC patients. FUT2 is an inhibitor of colorectal cancer metastasis which, when overexpressed, suppresses invasion and tumor dissemination in vitro and in vivo. FUT2 knock-out mice (FUT2^△IEC^ mice) develop AMO and DSS-induced tumors and promote EMT in colorectal cancers. FUT2-induced α-1,2 fucosylation impacts the ability of low-density lipoprotein receptor-related protein 1(LRP1) to suppress colorectal cancer invasion.

**Conclusions:**

Our study demonstrated that FUT2 induces α-1,2 fucosylation and inhibits EMT and metastasis of colorectal cancer through LRP1 fucosylation, suggesting that FUT2 may serve as a therapeutic target for colorectal cancer.

Video Abstract

**Supplementary Information:**

The online version contains supplementary material available at 10.1186/s12964-023-01060-0.

## Background

Colorectal cancer (CRC) is the third most common cancer and the second most common cause of cancer-related death worldwide [1]. Despite the recent approval of advanced therapies (i.e., surgery, chemotherapy and radiotherapy), metastatic CRC still occurs in more than 50% of patients who undergo resection [2]. Therefore, the identification of determinants of CRC metastasis is a key step toward effectively controlling tumor progression. Emerging evidence indicates that changes in glycosylation play a crucial functional role in tumor progression and metastasis [3]. Altered glycosylation is implicated in processes related to angiogenic signaling and endothelial cell adhesion [4, 5], which not only directly impact cell growth [6] and survival but also facilitate tumor-induced immunomodulation [7] and eventual metastasis [8, 9]. Glycosylation is a specific posttranslational modification that is mainly controlled by glycosyltransferases and glycosidases that orchestrate the addition of defined glycan structures to glycoproteins and/or lipids [10]. Glycosylation results in several functional changes to glycoproteins that confer unique features that are characteristic of cancer cells and the tumor microenvironment [11]. Aberrantly glycosylated proteins affect different steps of the metastasis process, including epithelial-mesenchymal transformation (EMT), migration, invasion/infiltration, and tumor cell extravasation [12]. One of the most common glycan modifications on proteins or lipids is the attachment of fucoses via the action of various glycosyltransferases [13]. Recent reports have shown that fucosylation is associated with cancer progression [14] and metastasis [15] in colorectal cancer. Aberrant fucosylation was shown to be associated with prometastatic and metastasis-suppressing functions in cancer [16, 17]. However, there is currently limited understanding of which enzymes and related fucosylation modifications are important for the progression and metastasis of colorectal cancer.

Fucosyltransferase 2 (FUT2) is a key enzyme that catalyzes the transfer of fucose to the terminal galactose of type 1 or type 2 disaccharide via *α*1,2-linkage [18]. Aberrant α-1,2 fucosylation is a hallmark of multiple types of cancers [19]. Recent reports have revealed important functions of FUT2 in cancers. For instance, a lectin microarray of melanoma identified FUT2 as an anti-metastatic factor, and silencing FUT2 promoted the invasion of melanoma cells [20]. Nevertheless, FUT2 overexpression increased cell migration and invasion in vitro and metastasis of breast cancer in vivo [21]. The role and molecular mechanisms of FUT2 in colorectal cancer remain largely unclear. Thus, identifying the role and target proteins of FUT2 as well as gaining insight into their biological functions in colorectal cancer are worthwhile.

## Materials and methods

### Cell lines and cell culture

The human colorectal cancer cell lines HCT116 and SW480 were purchased from American Type Culture Collection (ATCC). All CRC cell lines were authenticated by short tandem repeat analysis and were negative for mycoplasma. The cells were cultured with DMEM (Gibco, San Francisco, CA, USA) supplemented with 10% fetal bovine serum (Gibco) and 100 U/mL penicillin and streptomycin (Servicebio, Wuhan, China) at 37 °C in a humidified incubator in 5% CO_2_.

### Animal studies and clinical specimens

BALB/c nude mice and Wild-type C57BL/6 male mice were purchased from Beijing Huafukang Biological Co., Ltd. and housed under standard pathogen-free conditions in the Experimental Animal Center of Tongji Medical College, Huazhong University of Science & Technology. Pvillin-Cre recombinase transgenic C57BL/6 mice (Pvillin-Cre TG mice) and FUT2^flox/flox^ C57BL/6 mice (purchased from GemPharmatech Co. Ltd) were crossed and generated mice with FUT2 gene specifically deleted in intestinal epithelial cell (Pvillin-Cre + FUT2^*f*lox/flox^ mice, abbreviated as FUT2^△IEC^). 29 human samples were obtained from the Endoscopy Center of Union Hospital in 2020. All the samples were obtained with the patients’ informed consent, and the samples were processed histologically.

### Azoxymethane (AOM)- and dextran sulfate sodium (DSS)-induced colorectal cancer

On Day 0, WT and FUT2^△IEC^ mice were intraperitoneally (IP) injected with 10 mg/kg AOM working solution (1 mg/ml in isotonic saline, diluted from a 10 mg/ml stock solution in dH2O that was stored at − 20 deg C). On Day 7, mice were provided a continuous supply of 2.5% (2.5 g/100 ml) DSS solution in their drinking water for seven days. On Day 14, mice were again given standard drinking water for two weeks. The abovementioned DSS and water steps were repeated on Days 28 and 49 to provide a second and third cycle of DSS administration. Mice were sacrificed on Day 70, and samples were prepared for assessment.

### Virus construction and infection

Lentivirus for FUT2 overexpression and shRNA sequences targeting LRP1 were designed and constructed by Obio Technology Co. Ltd., Shanghai, China. OeRNA and shRNA lentivirus was added to the culture medium of GC cells with HitransG A (GeneChem, Shanghai, China), and puromycin was used to screen stable clones. The sequence targeting LRP1 was as follows: CATGCTGGACCTCTCCAATAA.

### RNA Extraction and real-time qPCR

Total RNA was extracted with RNAiso Plus reagent (Code No.: 9109, TAKARA, Japan) and reverse transcribed into cDNA using a verse Transcription system (Code No.: RR036A, TAKARA, Japan). Quantitative real-time polymerase chain reaction (qRT‒PCR) was performed using TB Green *Premix Ex Taq* (Tli RNaseH Plus) (Code No.: RR420A, TAKARA, Japan). The 2-^(ΔΔCt)^ method was used to calculate the relative abundance of RNA after normalization to GAPDH. For HCT116 and SW480 cells, cells were suspended and added to the 6-well plates (1.2 × 10^6^ cells/plate). After 24 h, cells grew to 80% fusion and the total RNA was extracted. The primers that were used for PCR are listed in Additional file 1: Table S1.

### Histology, immunohistochemistry (IHC) and immunofluorescence (IF) staining

Tissues were embedded and sectioned. After deparaffinization and rehydration, the sections were boiled for 30 min at 100 °C for antigen retrieval, treated with 3% hydrogen peroxide for 4 min and blocked by incubation with 10% goat serum for 30 min. Subsequent staining with primary antibodies was performed at room temperature for 60 min. After incubation with horseradish peroxidase (HRP)-conjugated secondary antibodies, the sections were washed in distilled water, treated with DAB, counterstained with hematoxylin, and dehydrated.

### Cell migration and invasion assay

Cell migration and invasion were measured using 24-well inserts (membrane pore size, 8 μm; Corning Life Sciences, MA, USA). For the migration assay, SW480 and HCT116 cells were suspended in serum-free medium (5 × 10^4^ cells/insert) and added to the upper chamber of the 24-well insert. Medium supplemented with 10% serum was added to the lower chamber. After incubation for 18 h, the cells that migrated were fixed in 4% paraformaldehyde and stained with 1% crystal violet for 10 min. For the invasion assay, chamber membranes were coated with Matrigel (BD Bioscience, San Jose, CA, USA).

### Quantitative proteomic analysis of tandem mass tags (TMT)

Colon tissues of mice within both groups(WT and FUT2^△IEC^ mice administered with AMO and DSS) were lysed into peptides. Peptides were labeled with the TMT reagent label and mixed into one sample. Then the mixed sample was separated by liquid chromatography, and the distillate was divided into 12 samples in series. An aliquot of each sample was loaded onto an analytical HPLC column using the auto sampler of an EASY-nLC 1000 HPLC. The results were searched against a decoy database and proteins.

### Immunoprecipitation

Immunoprecipitation was performed via a protein A immunoprecipitation kit (Roche) according to the manufacturer’s protocol. SW480 and HCT116 cells were suspended and added to the 10 cm dish. After 24 h, cells grew to 80% fusion and were lysed (lysis buffer: 50 mM Tris, 150 mM NaCl, 2 mM EDTA, protease inhibitor cocktail (Complete Mini, Roche), 0.5% Triton X-100) and incubated for 1 h on ice. The cell lysates containing the protein were incubated overnight with primary antibody-conjugated beads at 4 °C overnight. The beads were eluted with lysis buffer, and the proteins were resuspended in SDS sample buffer and resolved by Western blotting.

### Ulex europaeus agglutinin-I (UEA-I) enrichment assays

SW480 and HCT116 cells were transfected with a FUT2 overexpressing or control vector. Cells were suspended and added to the 10 cm dish. After 24 h, cells grew to 80% fusion and were were lysed with lysis buffer (50 mM Tris, 150 mM NaCl, 2 mM EDTA, protease inhibitor cocktail) containing 1% Nonidet P-40. The lysates (1000 μg) were mixed with 100 μl of biotin-conjugated UEA-I (0.1 µg/ml) and incubated with rotation at 4 °C overnight. Then, agarose coupled with streptavidin was added and incubated continuously for 4 h. The samples were extracted and separated by SDS‒PAGE and transferred to PVDF membranes. The membranes were incubated with LRP1, LAMB1 and Integrin β1 antibodies.

### Western blotting

HCT116 and SW480 cells were suspended and added to the 6-well plates (1.2 × 10^6^ cells/plate). After 24 h, cells grew to 80% fusion. Proteins were extracted from tissues and CRC cells in ice-cold RIPA lysis buffer containing phosphatase and protease inhibitor mixes (Beyotime Biotechnology, China) at 4 °C for 20 min. The proteins were separated by SDS‒PAGE and transferred to nitrocellulose membranes. The membranes were blocked using 10% milk. The membranes were then incubated overnight with primary antibodies at 4 °C, followed by washing three times in TBST before the addition of secondary antibodies and incubation for 1 h at room temperature. The membranes were further exposed to ECL with Millipore immobilon western chemilum HRP substrate.

### In vivo metastasis assay

HCT116 cells transduced with LV-FUT2 or LV-CON lentiviruses were harvested and resuspended at a concentration of 5 × 10^6^ cells per 100 μl and maintained on ice until injection. The cell suspensions were mixed with 100 μl Matrigel (Corning, BD) before injection. Two hundred microliters of cell/Matrigel (1:1) suspensions were then subcutaneously or peritoneally injected into four-week-old male BALB/c nude mice. Mice were sacrificed after 30 days, and peritoneal metastatic nodules were counted.

### Statistical analysis

All the experiments were repeated at least three times, and the results are expressed as the means ± SDs. All the statistical analyses were performed using GraphPad Prism software. Student’s t test or one-way analysis of variance (ANOVA) was used to analyze the data, and the chi-square test was used to analyze differences in other variables, as appropriate. A P value of < 0.05 was considered statistically significant for all datasets. (*p < 0.05, **p < 0.01, ***p < 0.001).

## Results

### FUT2 expression is downregulated in tumor tissues and inversely associated with the prognosis of CRC patients

**Fig. 1:**
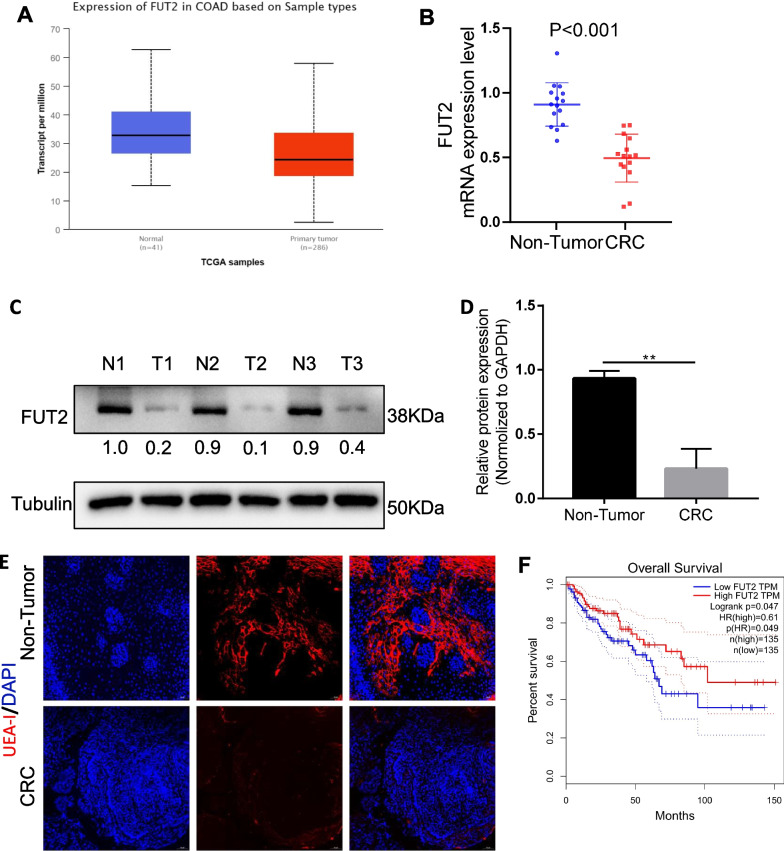
FUT2 and altered protein fucosylation are linked with prognosis of CRC patients. **A** FUT2 mRNA in CRC tissues and normal tissues in TCGA dataset. **B** FUT2 expression level in 29 CRC tissues and adjacent non-tumor tissues detected by qRT-PCR. **C** FUT2 expression level in CRC tissues and adjacent non-tumor tissues detected by western blotting. **D** Quantification of C. **p < 0.01. **E** Representative images of UEA-I lectin fluorescence of CRC tissues and adjacent non-tumor tissues. **F** The overall survival analysis was plotted using GEPIA for patients with CRC

Although glycosylation has been studied in colorectal cancer, these studies have focused on the N- and O-glycosylation of proteins [13]. It has been proposed that fucosylation deficiency leads to colitis and adenocarcinoma in mice [22], while it remains unclear whether α-1,2 fucosylation (FUT1, FUT2) is relevant to colorectal cancer. To examine the relationship between FUT2 and colorectal cancer (CRC), we performed bioinformatics analysis of FUT2 expression profiles in the TCGA database and found that FUT2 was significantly downregulated in CRC (Fig. 1A). Furthermore, we observed significantly decreased FUT2 mRNA expression levels in 29 CRC tissues relative to those in normal colorectal tissues (Fig. 1B). FUT2 protein expression was examined, and significantly lower expression levels of FUT2 were observed in CRC tissues than in corresponding normal tissues (Fig. 1C–D). Moreover, we observed a loss of UEA-I, specific for α-1,2 fucosylation, which is used as a global marker for fucose in CRC tissues (Fig. 1E). Gene Expression Profiling Interactive Analysis (GEPIA) showed that lower FUT2 expression was correlated with lower survival rates (Fig. 1F).

### FUT2 overexpression inhibits CRC cell EMT, migration and invasion in vitro

**Fig. 2:**
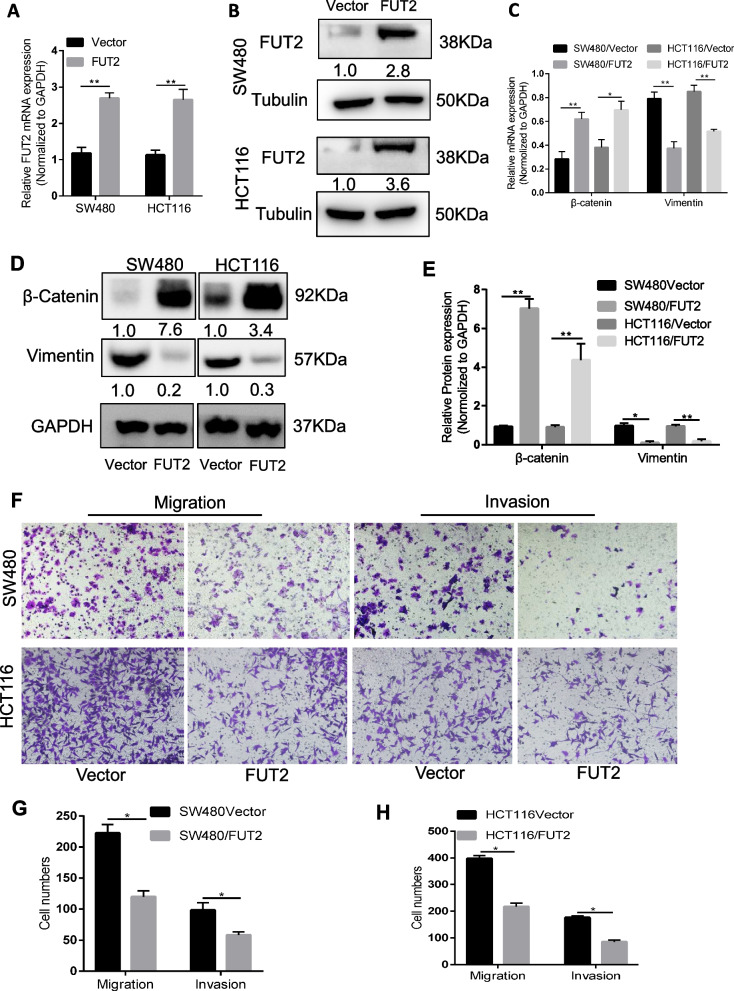
FUT2 overexpression inhibits CRC cell EMT and invasiveness in vitro. **A** FUT2 levels in SW480 and HCT116 cells after overexpression of FUT2 were assessed by qRT-PCR and western blot (**B**). **C** Quantification of B. *p < 0.05. **p < 0.01. **D** β-catenin and vimentin levels in SW480 and HCT116 cells after overexpression of FUT2 were assessed by western blot. **E** Quantification of D. **p < 0.01. **F** Transwell migration and invasion by SW480 and HCT116 cells transduced with FUT2 or Vector. **G**–**H** Quantification of F. *p < 0.05

Downregulation of α-1,2 fucosylation (FUT1, FUT2) and the resulting alteration in protein fucosylation were identified as features of metastatic behavior [20]. Keiichiro et al. demonstrated that FUT2 was significantly suppressed in colorectal cancer HT29 and DLD-1 cells undergoing epithelial-mesenchymal transition (EMT) [23]. EMT has been noted as a critical phenotypic alteration in metastatic cancer cells [24]. To further investigate the role of FUT2 in CRC metastasis, we overexpressed FUT2 in the CRC cell lines HCT116 and SW480, both of which express low endogenous levels of FUT2 (Additional file 2: Fig. S1). The stable overexpression of FUT2 was assessed by real-time qPCR and Western blotting (Fig. 2A–B).

Further analyses of the FUT2-overexpressing cells indicated a change in EMT. There was a clear increase in the surface expression of β-catenin and a decrease in the expression of the mesenchymal marker vimentin in FUT2-overexpressing cells (Fig. 2C–E). Moreover, FUT2 overexpression attenuated the migratory and invasive capacity of HCT116 and SW480 cells (Fig. 2F–H). Together, these observations suggest that FUT2 is a regulator of the EMT program and metastatic capacity in colorectal cancer cells.

### FUT2 overexpression decreases CRC EMT and metastasis in vivo

**Fig. 3:**
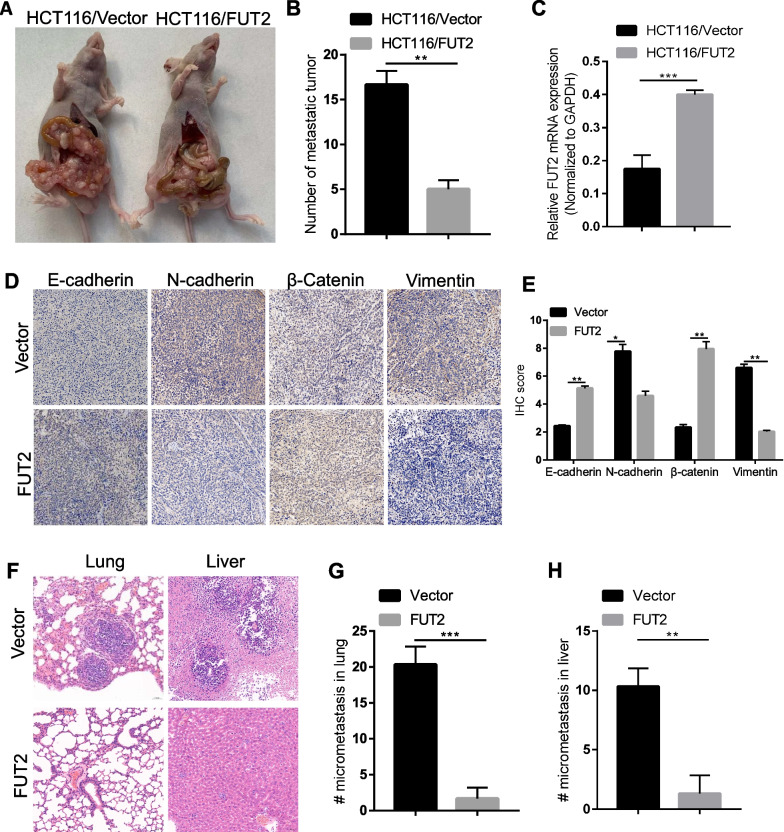
FUT2 overexpression decreases in vivo CRC EMT and metastasis. **A** Representative images of peritoneal metastases of nude mice injected with HCT116 cells transduced with LV-FUT2 or LV-NC. **B** Quantification of A. **p < 0.01. **C** FUT2 expression in tumors was assessed by western bloting. **D** Representative images of IHC staining with N-cadherin, E-cadherin, β-catenin and vimentin antibody in xenograft tumor sections from FUT2 overexpression cells. **E** Quantification of D. *p < 0.05. **p < 0.01. **F** H&E-stained images of mouse lungs and livers at end-point. **G**–**H** Quantification of F. **p < 0.01. ***p < 0.001

Next, we investigated whether FUT2 overexpression impairs aggressive behavior in vivo using a xenograft model of metastasis. HCT116 cells stably transduced with LV-FUT2 or LV-NC were transplanted intraperitoneally into nude mice, and tumor growth with the occurrence of peritoneal metastases was monitored. Similar to our in vitro observations, analyses of the peritoneal metastases revealed that the tumors derived from FUT2-overexpressing cells exhibited impaired aggression, as evidenced by fewer peritoneal metastatic nodules (Fig. 3A–B). The observation of upregulated FUT2 transcripts by qRT‒PCR confirmed the effective overexpression of FUT2 by both LVRNAs in tumors (Fig. 3C). IHC staining of sections of the xenograft tumors derived from FUT2-overexpressing cells revealed decreased expression of N-cadherin and vimentin as well as increased expression of E-cadherin and β-catenin (Fig. 3D–E). Mice injected with FUT2-overexpressing HCT116 cells exhibited significantly reduced metastases in the lungs and livers (Fig. 3F–H). Together, these data demonstrate that FUT2 overexpression inhibits the metastatic capacity of CRC in vivo.

### FUT2-knockout mice develop AMO- and DSS-induced tumors

**Fig. 4:**
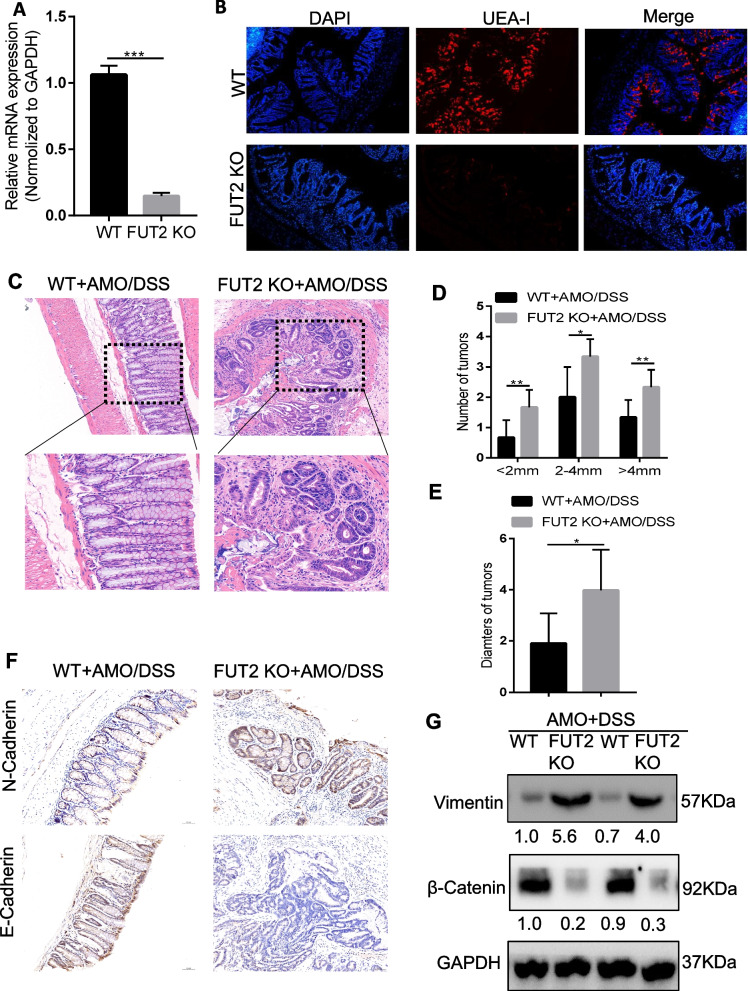
FUT2 knock-out mice develop AMO and DSS-induced tumors. **A** FUT2 levels in colon lysates of WT mice and FUT2 knock-out mice were detected by qRT-PCR. **B** Representative images of UEA-I lectin fluorescence staining revealed α-1,2 fucosylation in WT mice and Fut2^△IEC^ mice. **C** Representative images of H&E-stained colon sections after AMO and DSS administered. **D**–**E** Quantification of C. *p < 0.05. **p < 0.01. **F** Representative images of IHC staining with N-cadherin and E-cadherin in colon lysates in colon sections in WT mice and FUT2 knock-out mice after AMO and DSS administered. **G** FUT2 knock-out increased N-cadherin and vimentin, as shown by Western blotting

Intestinal-specific FUT2-knockout mice (FUT2^△IEC^ mice) were generated by hybridizing FUT2-knockout mice with Villin-CRE transgenic mice. qRT‒PCR analysis of colon lysates from *WT* and *fut2* − / − mice confirmed the reduction or absence of FUT2 expression (Fig. 4A). UEA-I lectin fluorescence staining confirmed the decrease in α-1,2 fucosylation in FUT2^△IEC^ mice (Fig. 4B). We then examined whether FUT2 knockout led to the development of DSS- and AMO-induced tumors. Mice within both groups (WT and FUT2^△IEC^ mice) were subdivided into two groups and treated with or without AMO and DSS. FUT2^△IEC^ mice developed significantly increased established colon tumors in comparison to WT mice 90 days after AMO and DSS treatment (Fig. 4C–E). To better study the role of FUT2 in colorectal cancer, colons were analyzed for the expression of EMT markers. IHC staining and Western blotting confirmed higher EMT in FUT2^△IEC^ mice treated with AMO and DSS than in WT mice treated with AMO and DSS, as evidenced by increased vimentin expression and decreased β-catenin expression (Fig. 4F–G). In summary, our results demonstrate that FUT2-knockout mice develop more AMO- and DSS-induced tumors and exhibit greater colorectal cancer EMT.

### 1.2-fucosylated glycoproteins reveal regulators of EMT and metastasis

**Fig. 5:**
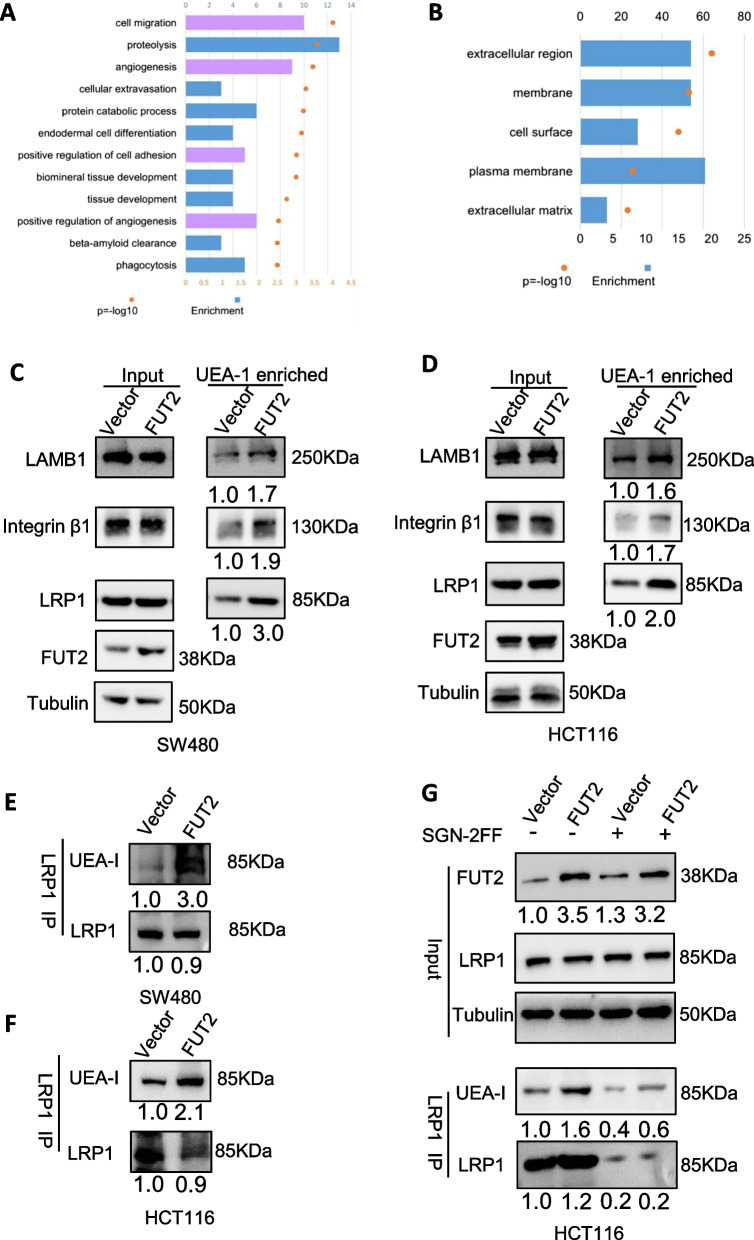
1.2- fucosylated glycoproteins reveals regulators of EMT and metastasis. **A** GO enrichment analysis of N-fucosylated proteins in WT and Fut2^△IEC^ mice based on biological processes. **B** GO enrichment analysis (category of functional categories) of N-fucosylated proteins in WT and Fut2^△IEC^ mice. **C** Validation of 1,2 fucosylation in proteins bound with UEA-I related to migration in SW480 cells. **D** Validation of 1,2 fucosylation in proteins bound with UEA-I related to migration in HCT116 cells. **E** IP of LRP1 from cell lysates of SW480 cells transduced with LV-FUT2 or LV-NC. **F** IP of LRP1 from cell lysates of HCT116 cells transduced with LV-FUT2 or LV-NC. **G** LRP1 immunoprecipitation from cell lysates of HCT116 cells transfected with LV-FUT2 or LV-NC. Anti-LRP1 immunoprecipitates were treated with or without SGN-2FF and blotted with UEA-I or α-LRP1.

FUT2 can generate α-1,2 fucosylated structures both at the termini of N-acetyllactosamine and on galactose linked with N-acetylgalactosamine, which are epitopes recognized by UEA-I and TJA-II/SNA-II lectins [20, 25]. To identify α-1,2 fucosylated proteins that could mediate the effects of FUT2 dysregulation on colorectal cancer, we performed a quantitative proteomics study of total protein and N-glycosylated TMT markers from colon tissues of mice in both groups (WT and FUT2^△IEC^ mice). A total of 324 candidate downregulated α-1,2 fucosylated proteins were identified in the proteomics study of N-glycosylation in FUT2^△IEC^ mice. Enrichment (Gene Ontology; GO) analysis of genes was performed and revealed enrichment in biological processes relevant to metastasis, such as cell migration and angiogenesis. The proteins that bound to UEA-I and were related to migration include laminin β1 (LAMB1), laminin β2 and laminin γ1, integrin-αV, integrin β1, signal-regulatory protein alpha (Sirpa) and Pro-low-density lipoprotein receptor-related protein 1 (LRP1) (Additional file 3: Table S2 and Fig. 5A–B). The glycosylation state of these proteins in colorectal cancer cells was further verified by UEA-I lectin enrichment followed by Western blotting. The results demonstrated increased integrin β1, LAMB1 and LRP1 levels in FUT2-overexpressing SW480 and HCT116 cells compared to LV-CON cells, consistent with lower α-1,2 fucosylation on those proteins in FUT2^△IEC^ mice, while there was no difference in the expression of these proteins in the input samples (Fig. 5C–D). Additionally, immunoprecipitation (IP) of LRP1 followed by UEA-I blotting showed increased UEA-I binding to LRP1 proteins in FUT2-overexpressing CRC cells (Fig. 5E–F). After SGN-2FF treatment, the observed differences disappeared because SGN-2FF removed all forms of fucosylation (Fig. 5G). Overall, our results suggest that LRP1 was α-1,2 fucosylated, which was consistent with our quantitative proteomics study.

### LRP1 is a major mediator of the suppressive role of FUT2 in metastasis

**Fig. 6:**
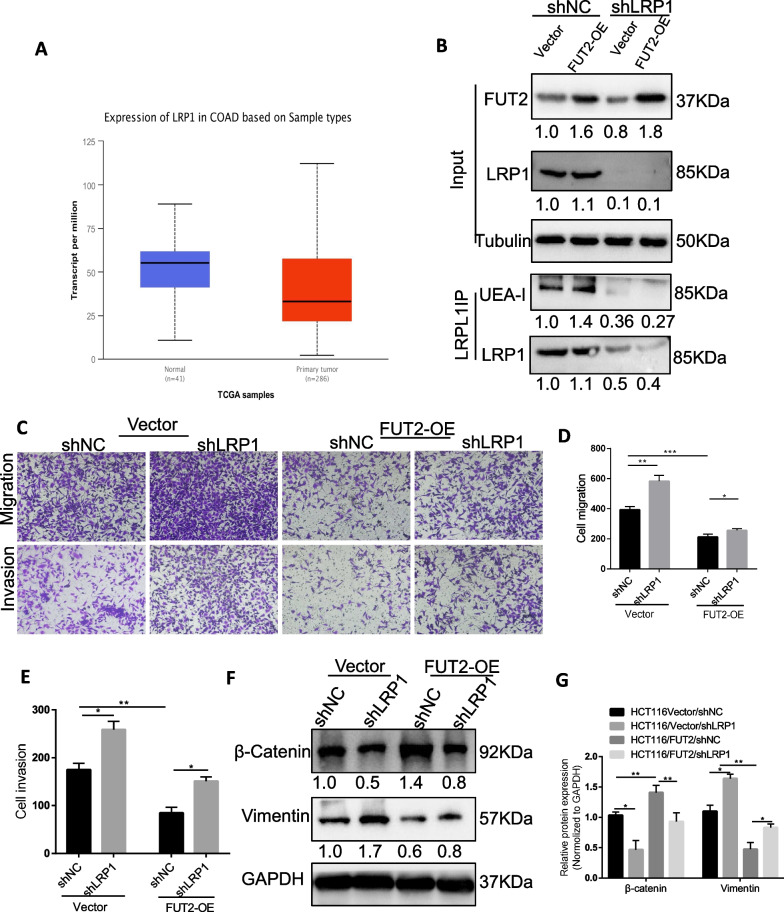
LRP1 is a major mediator of the suppressing metastatic role by FUT2. **A** Downregulation of LRP1 mRNA in CRC tissues than normal tissues in TCGA dataset. **B** LRP1 IP on cell lysates of HCT116 cells stably overexpressing FUT2 or control vector and transfected with NC or LRP1 shRNA. Anti-LRP1 immunoprecipitates were blotted with UEA-I or α-LRP1. **C** Transwell migration and invasion assays by HCT116 cells stably overexpressing FUT2 or control vector and transfected with NC or LRP1 shRNA. **D**–**E** Quantification of C. **F** β-catenin and vimentin levels in HCT116 cells stably overexpressing FUT2 or control vector and transfected with NC or LRP1 shRNA were assessed by western blot. **G** Quantification of F

LRP1 is a highly glycosylated cysteine-rich protein. Numerous studies have suggested a role for LRP1 in the regulation of cell invasion and migration in several cancers [26, 27], and it may suppress CRC progression [28]. Furthermore, bioinformatics analysis of LRP1 expression profiles in the TCGA database indicated that LRP1 was expressed at lower levels in CRC than in noncancerous colon tissues (Fig. 6A). Therefore, it is a possible candidate for the effects of FUT2 in suppressing CRC invasion. We next investigated whether LRP1 silencing accelerated cell invasion and migration in HCT116 cells overexpressing FUT2. Our data suggested that LRP1 exhibits increased ɑ-1,2 fucosylation in FUT2-overexpressing HCT116 cells (Fig. 6B). Furthermore, FUT2 overexpression increased ɑ-1,2 fucosylation and neutralized the pro-invasive effects of LRP1 silencing (Fig. 6C–E). In addition, silencing LRP1 counteracted the inhibitory effects of FUT2 overexpression on EMT (Fig. 6F–G). Our data suggest that decreased ɑ-1,2 fucosylation is critical for the effects of LRP1 in suppressing the invasive phenotype of CRC cells.

## Discussion

Modifications of glycosylation at the surface of tumor cells and tissue induce unique features that are characteristic of cancer cells and the tumor microenvironment [13]. Several studies have revealed that aberrant glycosylation plays important roles in tumor progression and malignant transformation [29]. One of the most common modifications of glycans is the attachment of fucoses via the action of various fucosyltransferases [13]. Nakayama et al. described a novel metastatic pathway that is dependent on the loss of fucosylation in colorectal cancer [15]. Recent studies indicate that carcinogenesis in a subset of colorectal cancer in mice occurs due to a molecular mechanism driven by fucosylation deficiency [22]. There is currently limited understanding of which enzymes and related fucosylation modifications are important in the progression and metastasis of colorectal cancer. The present study demonstrates how ɑ-1,2 fucosylation, mediated by FUT2, impacts CRC progression and metastasis.

FUT2, an enzyme governing epithelial α-1,2 fucosylation, is associated with various human disorders [30]. FUT2 mediates the addition of L-fucose via an α-1–2 linkage to the terminal β-D-galactose residues of mucosal glycans, including type 1 or 2 N-acetyllactosamine [31]. Recent reports have shown that FUT2 is preferentially expressed in epithelial cells (ECs) of the gastrointestinal tract [32] and that α-1,2 fucose on enterocytes is specifically regulated by FUT2 [33]. Several studies have revealed that defects in epithelial FUT2 predispose individuals to several diseases [30], including inflammatory bowel disease (IBD, especially Crohn’s disease) [34], acute and chronic inflammatory disorders such as type I diabetes [35], and chronic pancreatitis [36]. However, the role of changes in fucosylation induced by FUT2 in colorectal cancer remains unclear. Our results revealed a loss of α-1,2 fucosylation (UEA-I) in human colorectal cancer tissues. These data are consistent with a previous study showing that fucosylation deficiency in mice leads to colitis and adenocarcinoma [22]. We observed that FUT2 knockout resulted in increased DSS-induced colon tumors and EMT in FUT2△IEC mice, indicating the inhibitory effects of FUT2 and α-1,2 fucosylation on the progression and metastasis of colorectal cancer. Lau et al. identified a role of α-1,2 fucose in suppressing the metastatic potential of melanoma and correlated it with higher survival rates [37]. Our results are consistent with this finding and highlight the inhibitory effects of FUT2 on EMT in colorectal cancer.

Next, we focused functional and mechanistic studies of FUT2. FUT2 was significantly suppressed during the EGF- or bFGF-triggered EMT of colorectal cancer cells [23]. However, the role of this enzyme and the α-1,2 fucosylation it causes in colorectal cancer is still unknown. FUT2 overexpression in the colorectal cancer cell lines HCT116 and SW480 attenuated their migratory and invasive capacities. This is in contrast to results in breast cancer cell lines [21] and lung adenocarcinoma cells [38], where FUT2 enhances cell migration and invasion. Overexpressing FUT2 reduced EMT in colorectal cancer cells, suggesting that α-1,2 fucosylation may inhibit EMT. Similar results were obtained in vivo: FUT2 overexpression reduced metastatic dissemination of colorectal cancer cells to the peritoneum and inhibited EMT in vivo. Together with these results, our present study demonstrates that loss of FUT2 and α-1,2 fucosylation may promote EMT and metastasis in colorectal cancer.

One of the most important aspects of our study is related to the involvement of LRP1 in the regulation of EMT and metastasis by FUT2. We identified LRP1 as a mediator of the invasive-inhibiting effects of FUT2 using a proteomics study and immunoprecipitation. LRP1 is a cell surface receptor involved in invasion and neovascularization, processes that drive tumor progression and metastasis [39]. Previous studies have shown that LRP1 expression is significantly lower in colon adenocarcinoma cells than in colon mucosa and stromal cells [40] and is associated with worse colorectal cancer outcomes [28]. Consistent with these previous studies, we found that LRP1 was downregulated in CRC tissues compared to nontumor tissues. Overexpressing FUT2 reduced the EMT and invasion of colorectal cancer cells. However, these invasive-inhibiting effects were mostly abrogated by silencing LRP1. Our results demonstrated that α-1,2 fucosylation was crucial for the inhibition of EMT and invasion by LRP1 in colorectal cancer cells.

Taken together, our results highlight the inhibitory potential of FUT2 in colorectal cancer. In addition, we provide a number of solid arguments for a regulatory role of FUT2 and the α-1,2 fucosylation it causes in the function of LRP1. These data may not only enrich our knowledge of fucosylation but also suggest a strategy for therapeutic intervention in colorectal cancer.

## Conclusions

Our study demonstrated that FUT2 induces α-1,2 fucosylation and inhibits the EMT and metastasis of colorectal cancer through LRP1 fucosylation, suggesting that FUT2 may serve as a therapeutic target for colorectal cancer (Additional file 4).

## Supplementary Information

**Additional file 1**. Table S1. Primers for qRT-PCR.

**Additional file 2**. Figure S1. Expression of FUT2 gene in CRC cell lines.

**Additional file 3**. Table S2. Mass spectrometric analysis of N-glycosylated TMT proteins in migration category of GO analysis from colon tissues of mice in WT and FUT2^△IEC^ mice.

**Additional file 4**. Uncropped images of western blot analysis.

## Data Availability

The datasets used and/or analysed during the current study are available from the corresponding author on reasonable request.

## Abbreviations

FUT2: Fucosyltransferase 2
CRC: Colorectal cancer
LRP1: Low-density lipoprotein receptor-related protein 1
EMT: Epithelial-mesenchymal transformation
qRT-PCR: Real-Time qPCR
IHC: Immunohistochemistry
IF: Immunofluorescence
AOM: Azoxymethane
DSS: Dextran sulfate sodium
ATCC: American type culture collection
GEPIA: Gene expression profiling interactive analysis
UEA-I: Ulex europaeus agglutinin-I
WT: Wild type
ECs: Epithelial cells
EGF: Epidermal growth factor
bFGF: Basic fibroblast growth factor
