# Supplementary material for: FUT2 inhibits the EMT and metastasis of colorectal cancer by increasing LRP1 fucosylation
Source: Cell Commun Signal. 2023 Mar 27;21:63. doi: 10.1186/s12964-023-01060-0 (PMC10041739; doi:10.1186/s12964-023-01060-0)
Supplement: Supplementary file 2 — Additional file 1. Table S1. Primers for qRT-PCR. [file 12964_2023_1060_MOESM2_ESM.docx]

Supplementary Table S1. Primer for RT-PCR

| **Number** | **Primer for qRT-PCR** | **Forward primer** | **Reverse primer** |
| --- | --- | --- | --- |
| 1 | FUT2-mouse | CAGGATGAACGGTCGGCTTGC | TTCTGGCTGTGTCGCTGTGTAAC |
|  | FUT2-human | ATCATGACCATTGGGACGTT | GTGCTTGAGTAAGGGGGACA |
| 2 | β-catenin-human | TCTTGCCCTTTGTCCCGCAAATCA | TCCACAAATTGCTGTGTCCCA |
| 3 | Vimentin-human | CCTGCAATCTTTCAGACAGG | CTCCTGGATTTCCTCTTCGT |
| 4 | GAPDH-mouse | TGCACCACCAACTGCTTAG | GGATGCAGGGATGATGTTC |
| 5 | GAPDH-human | GGAGCGAGATCCCTCCAAAAT | GGCTCTTGTCATACTTCTCATGG |
