# Supplementary figures and images for: FUT2 inhibits the EMT and metastasis of colorectal cancer by increasing LRP1 fucosylation
Source: Cell Commun Signal. 2023 Mar 27;21:63. doi: 10.1186/s12964-023-01060-0 (PMC10041739; doi:10.1186/s12964-023-01060-0)

**Supplementary Figure S1.** Expression of FUT2 gene in CRC cell lines.

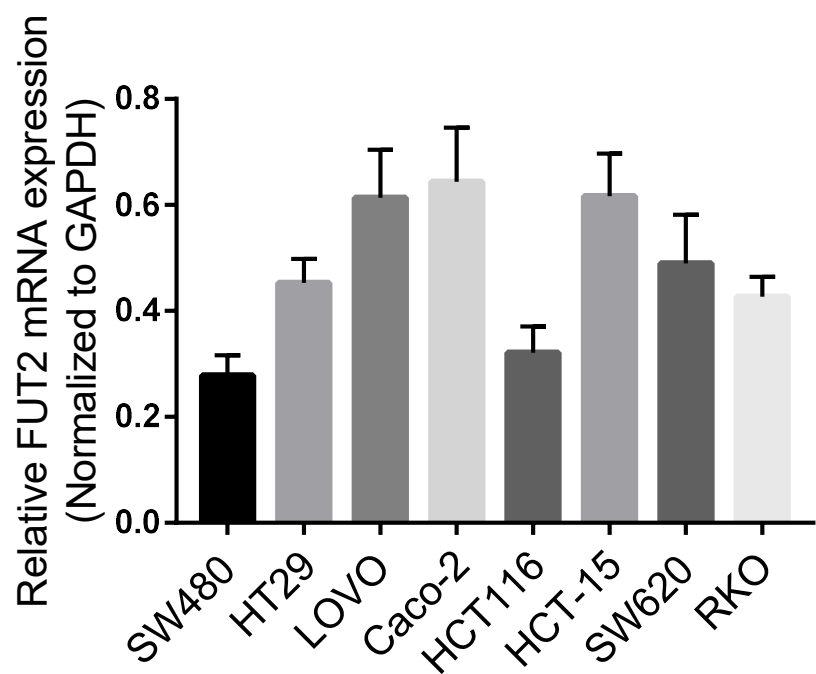

Supplement: Supplementary file 3 — Additional file 2. Figure S1. Expression of FUT2 gene in CRC cell lines. [file 12964_2023_1060_MOESM3_ESM.pdf]
