# Supplementary material for: FUT2 inhibits the EMT and metastasis of colorectal cancer by increasing LRP1 fucosylation
Source: Cell Commun Signal. 2023 Mar 27;21:63. doi: 10.1186/s12964-023-01060-0 (PMC10041739; doi:10.1186/s12964-023-01060-0)
Supplement: Supplementary file 4 — Additional file 3. Table S2. Mass spectrometric analysis of N-glycosylated TMT proteins in migration category of GO analysis from colon tissues of mice in WT and FUT2△IEC mice. [file 12964_2023_1060_MOESM4_ESM.docx]

**Supplementary Table2.** Mass spectrometric analysis of N-glycosylated TMT proteins in migration category of GO analysis from colon tissues of mice in WT and FUT2^△IEC^ mice.

|  | Description | Gene name | Score | Ratio | P value | Regulated Type |
| --- | --- | --- | --- | --- | --- | --- |
| P16406 | Glutamyl aminopeptidase OS=Mus musculus OX=10090 GN=Enpep PE=1 SV=1 | Enpep | 142.1 | 0.408 | 2.67781E-05 | Down |
| P43406 | Integrin alpha-V OS=Mus musculus OX=10090 GN=Itgav PE=1 SV=2 | Itgav | 133.48 | 0.739 | 0.002062348 | Down |
| P09055 | Integrin beta-1 OS=Mus musculus OX=10090 GN=Itgb1 PE=1 SV=1 | Itgb1 | 289.75 | 1.49 | 0.000236206 | Down |
| Q3UV74 | Integrin beta-2-like protein OS=Mus musculus OX=10090 GN=Itgb2l PE=1 SV=1 | Itgb2l | 149.35 | 0.088 | 5.54663E-07 | Down |
| P02469 | Laminin subunit beta-1 OS=Mus musculus OX=10090 GN=Lamb1 PE=1 SV=3 | Lamb1 | 134.06 | 0.27 | 2.60879E-06 | Down |
| Q91ZX7 | Prolow-density lipoprotein receptor-related protein 1 OS=Mus musculus OX=10090 GN=Lrp1 PE=1 SV=1 | Lrp1 | 206.58 | 0.231 | 2.17471E-06 | Down |
| Q61292 | Laminin subunit beta-2 OS=Mus musculus OX=10090 GN=Lamb2 PE=1 SV=2 | Lamb2 | 137.98 | 0.389 | 0.000113892 | Down |
| P02468 | Laminin subunit gamma-1 OS=Mus musculus OX=10090 GN=Lamc1 PE=1 SV=2 | Lamc1 | 251.84 | 0.226 | 5.89905E-06 | Down |
| P97797 | Tyrosine-protein phosphatase non-receptor type substrate 1 OS=Mus musculus OX=10090 GN=Sirpa PE=1 SV=2 | Sirpa | 88.948 | 0.544 | 0.00125089 | Down |
| O88307 | Sortilin-related receptor OS=Mus musculus OX=10090 GN=Sorl1 PE=1 SV=3 | Sorl1 | 155.42 | 0.642 | 0.000704075 | Down |
