# Supplementary material for: FUT2 inhibits the EMT and metastasis of colorectal cancer by increasing LRP1 fucosylation
Source: Cell Commun Signal. 2023 Mar 27;21:63. doi: 10.1186/s12964-023-01060-0 (PMC10041739; doi:10.1186/s12964-023-01060-0)

Figure 2B

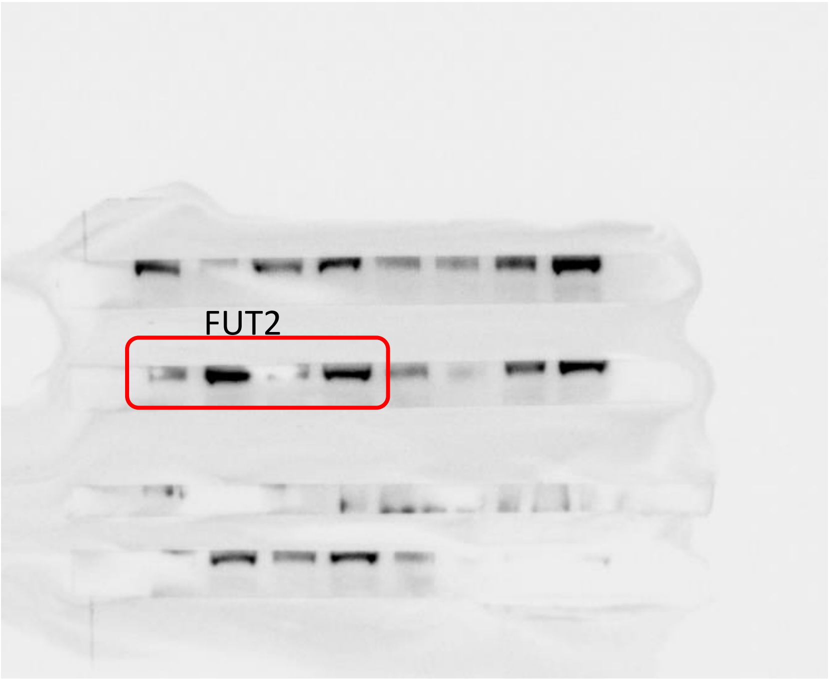

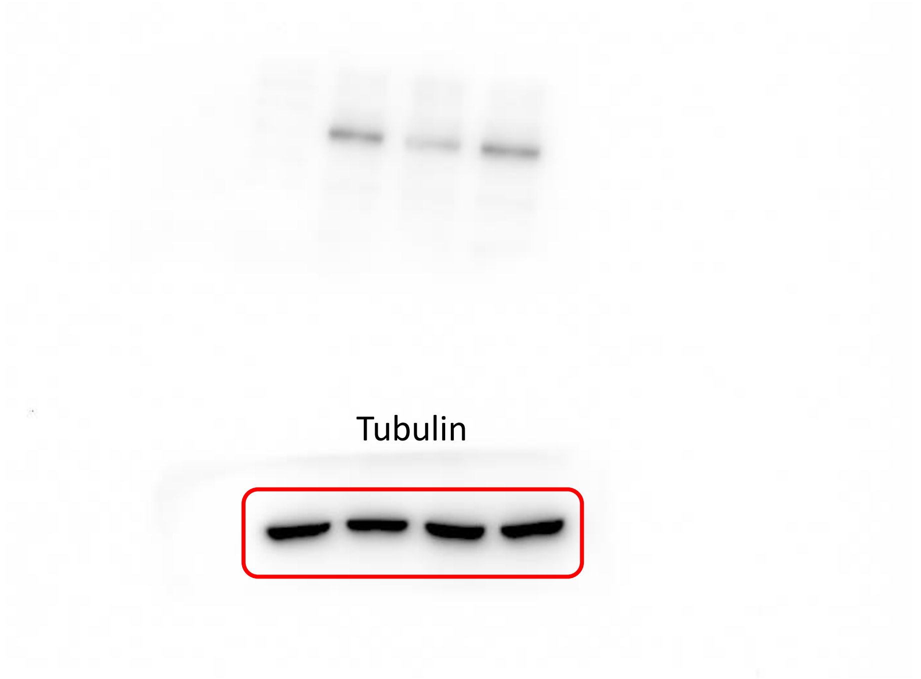

Figure 2D

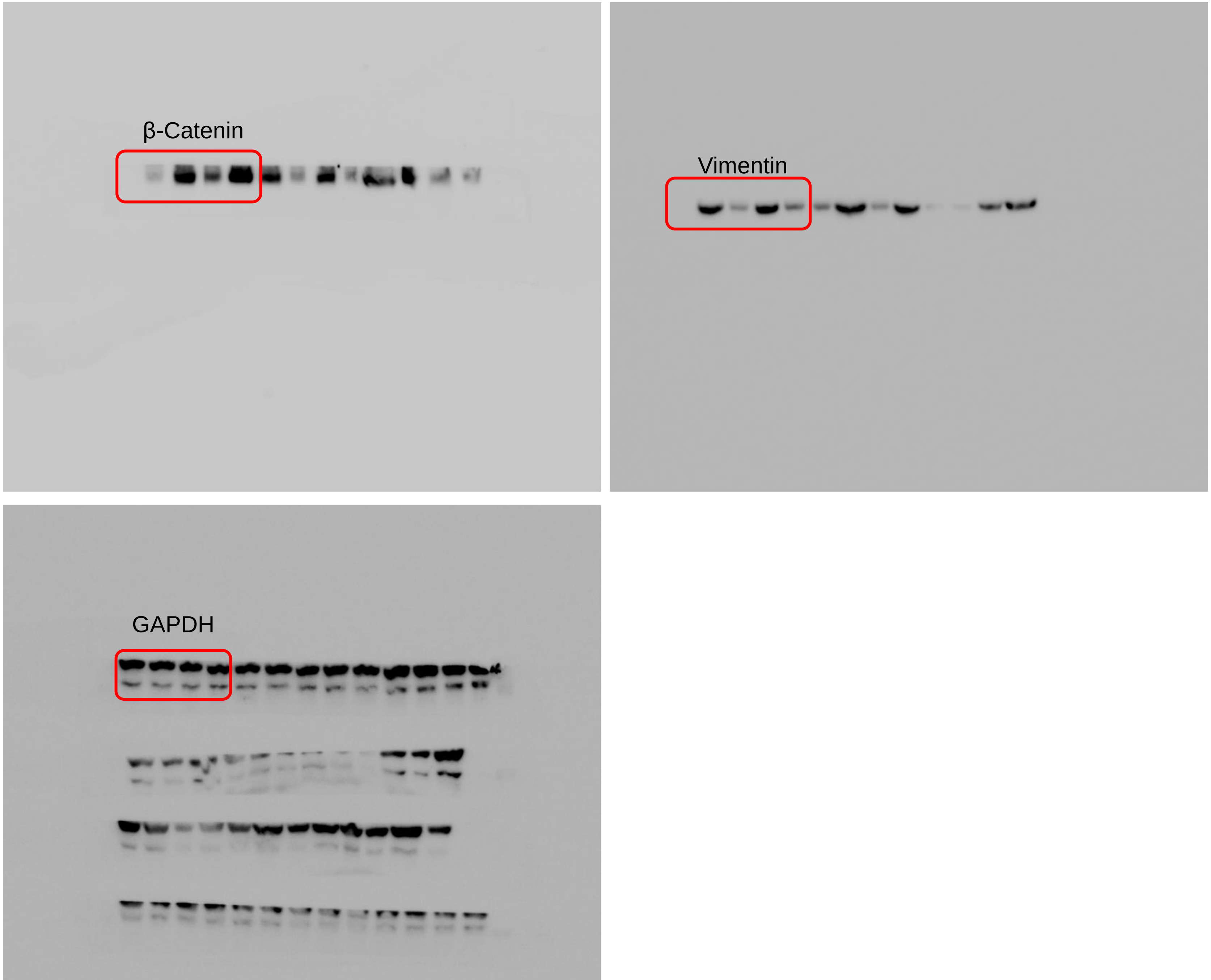

Figure 4G

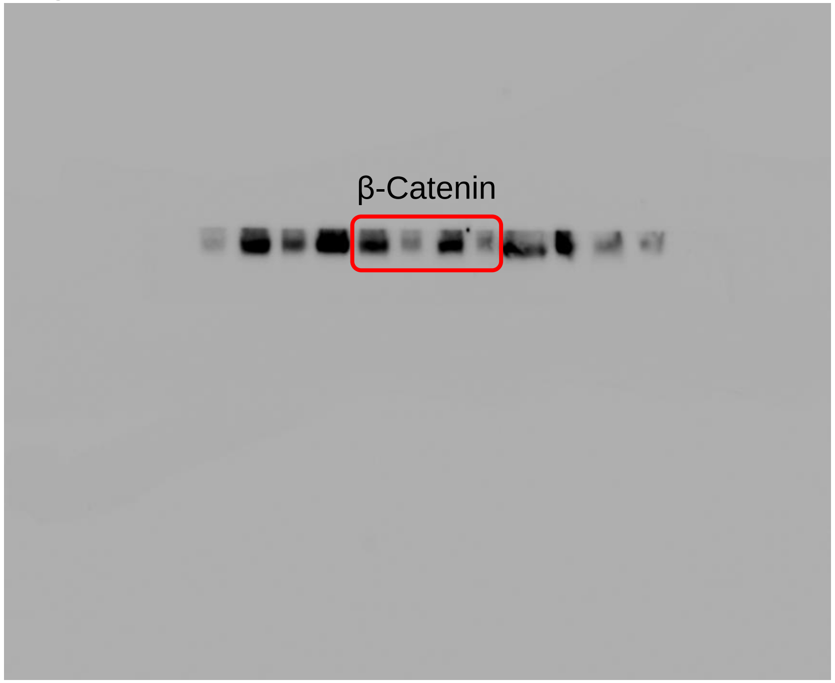

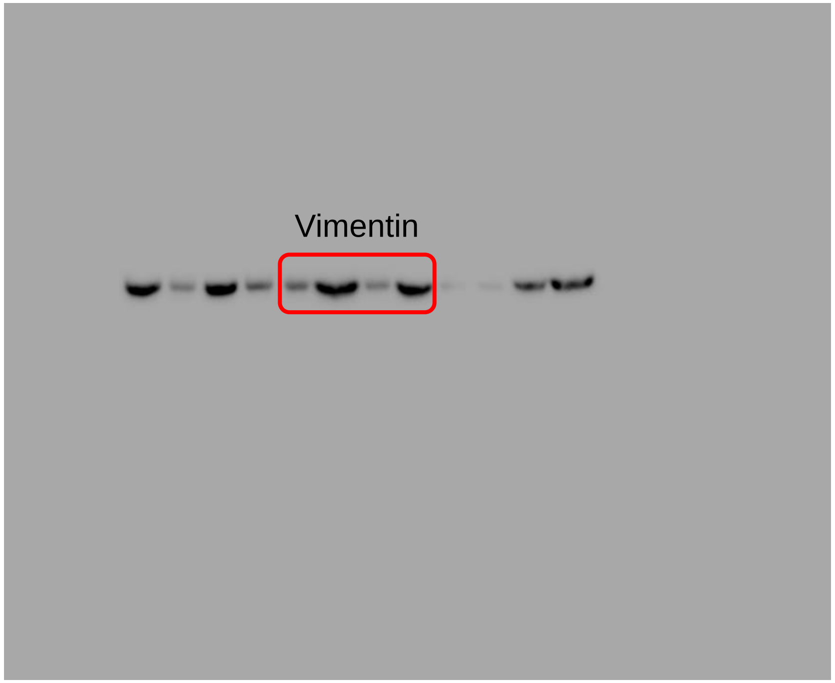

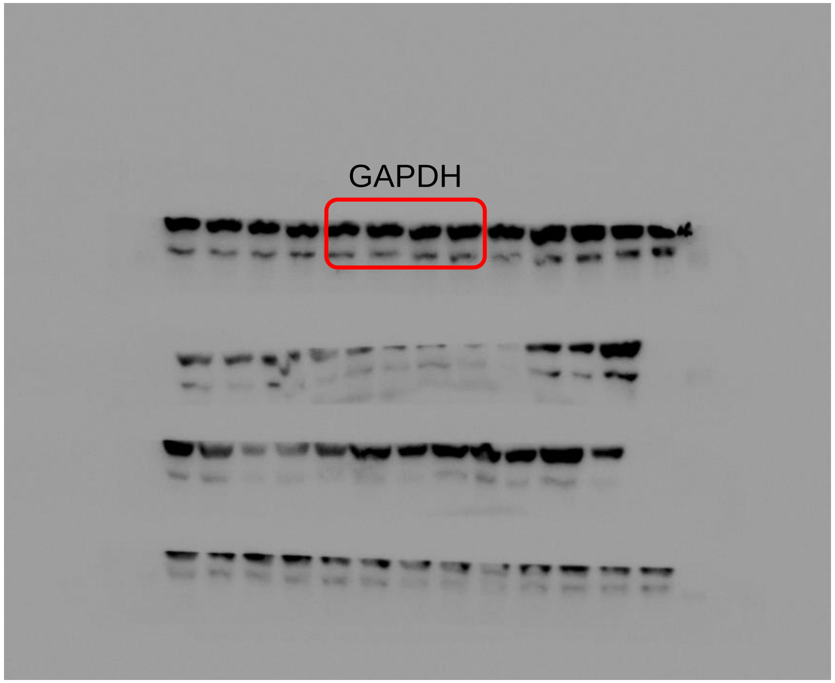

Figure 5B

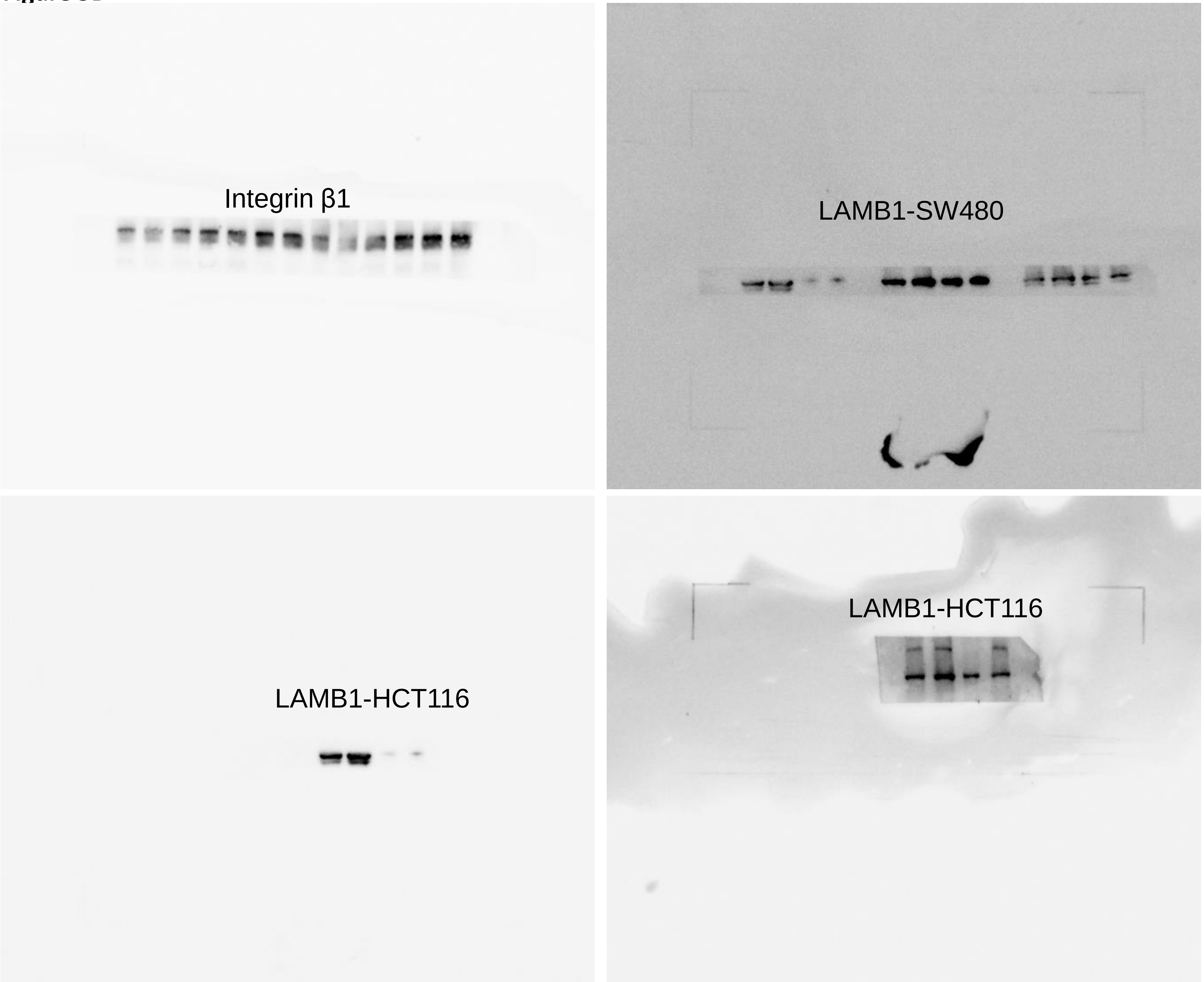

Figure 5B

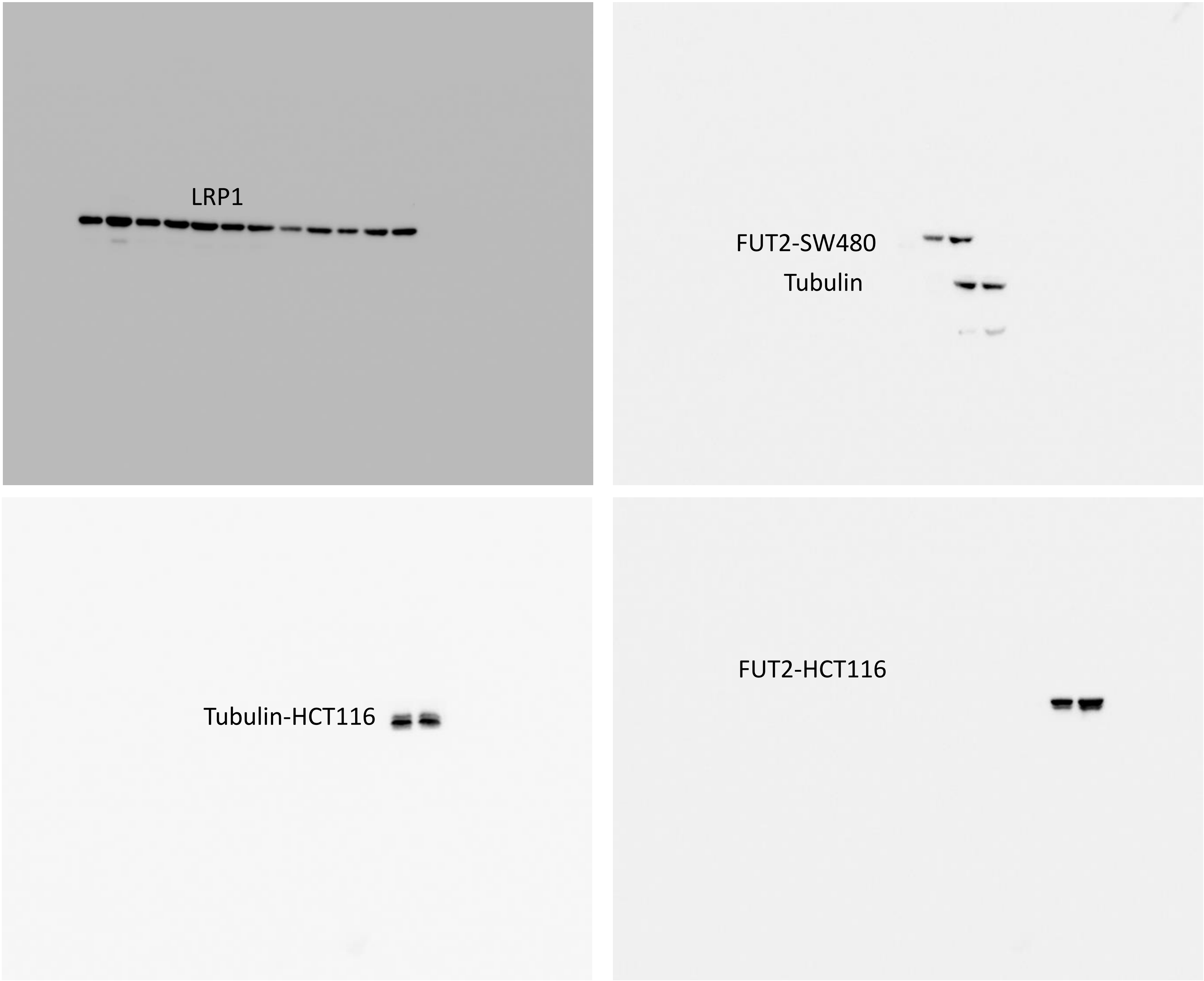

Figure 5E-F

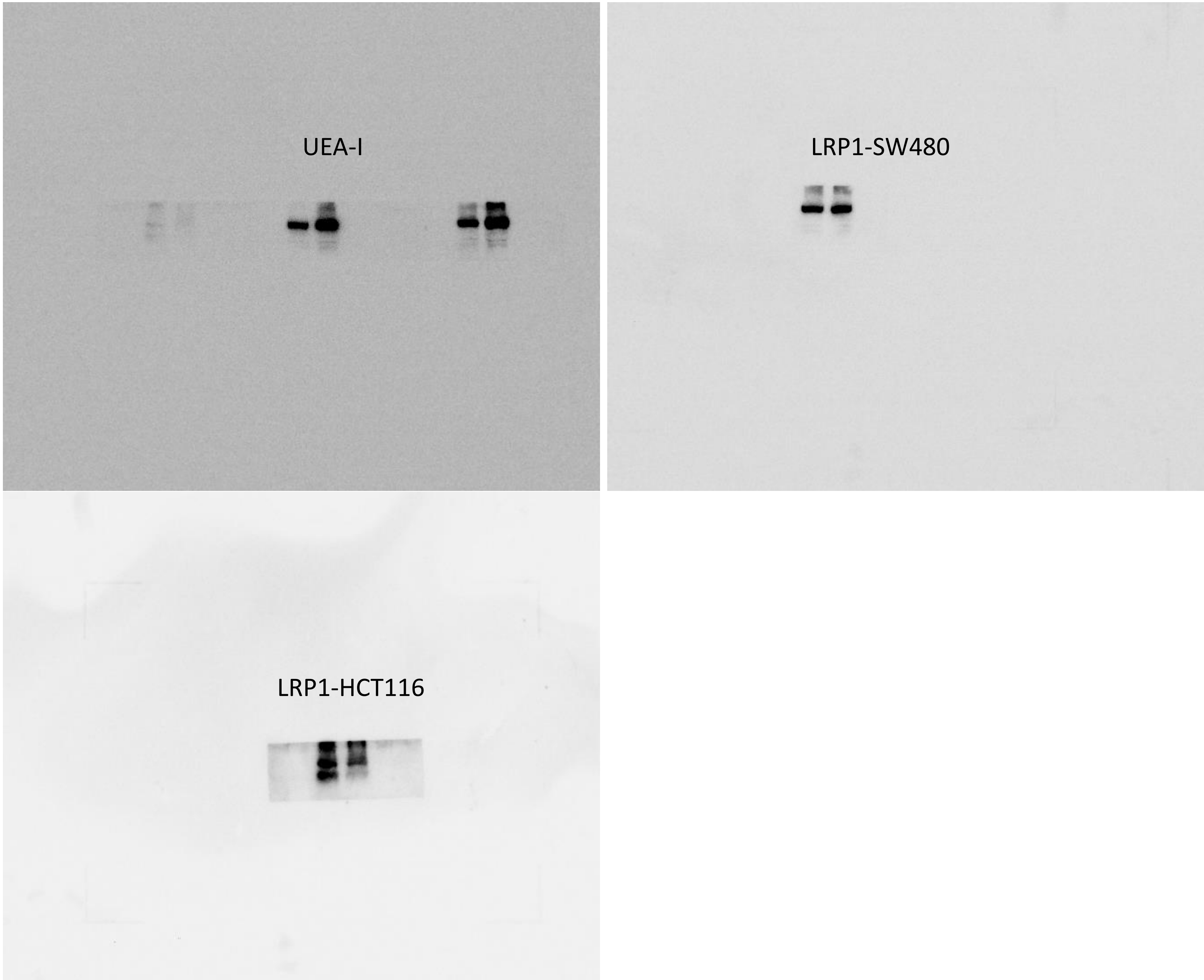

Figure 5G

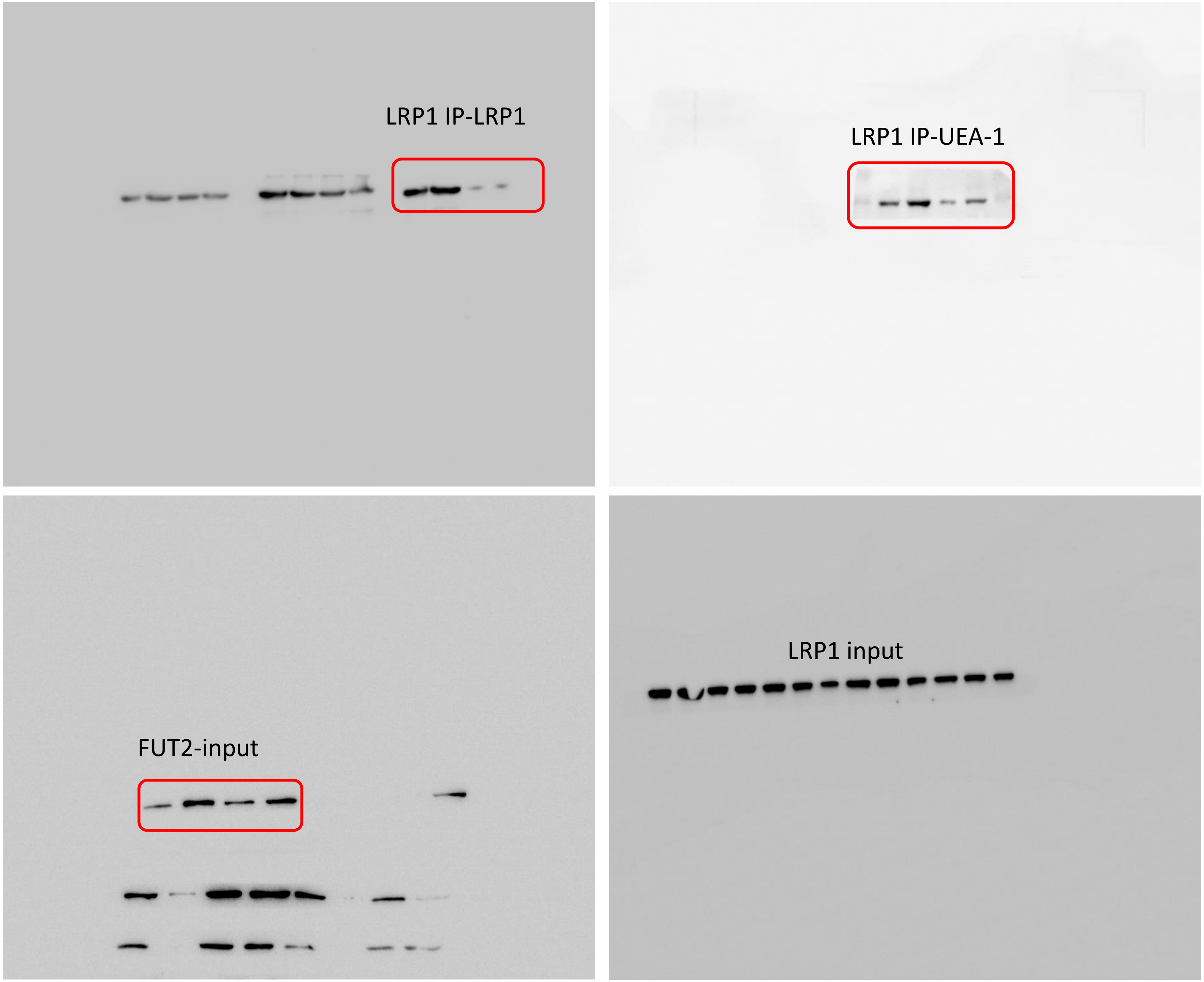

Figure 5G

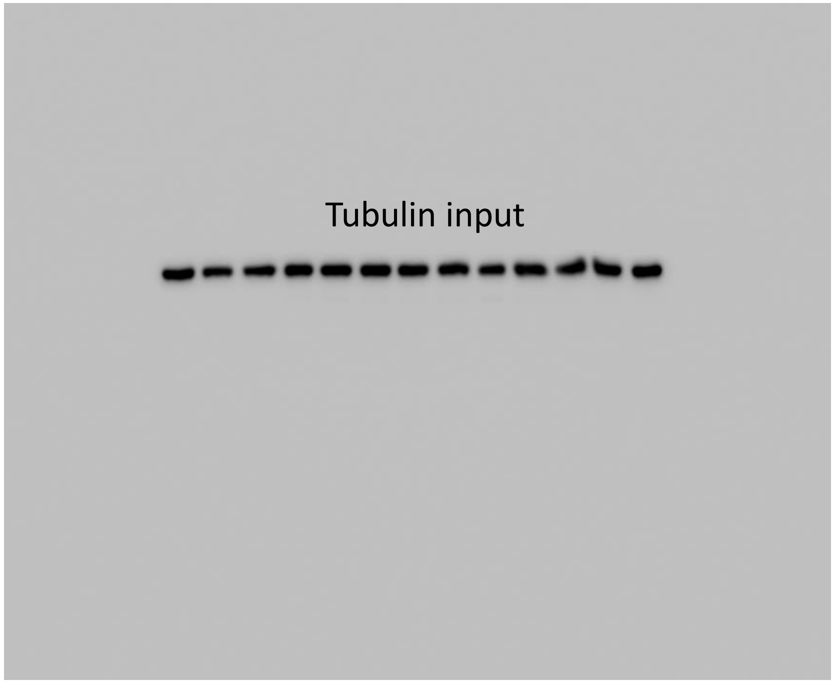

Figure 6B

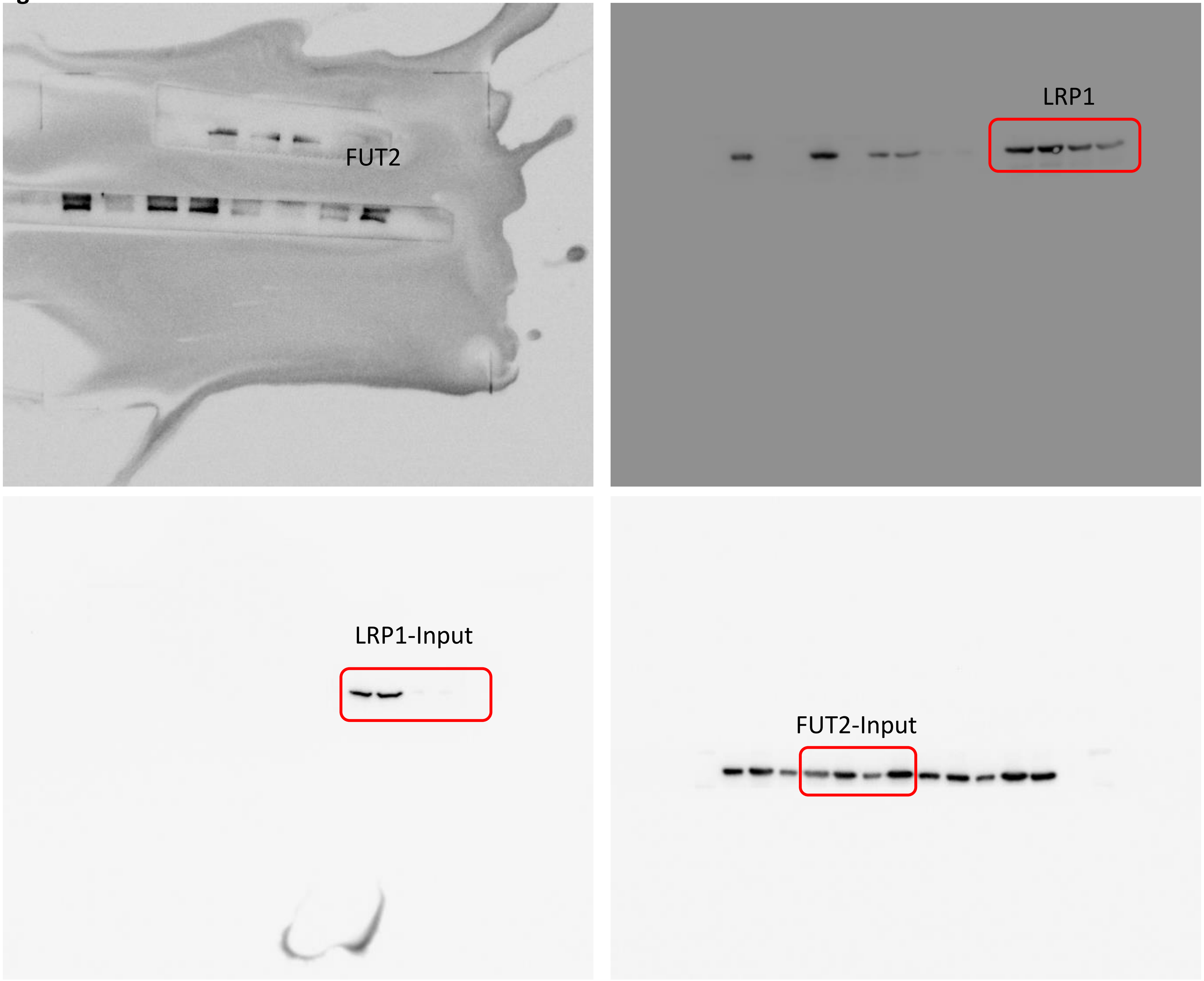

Figure 6B

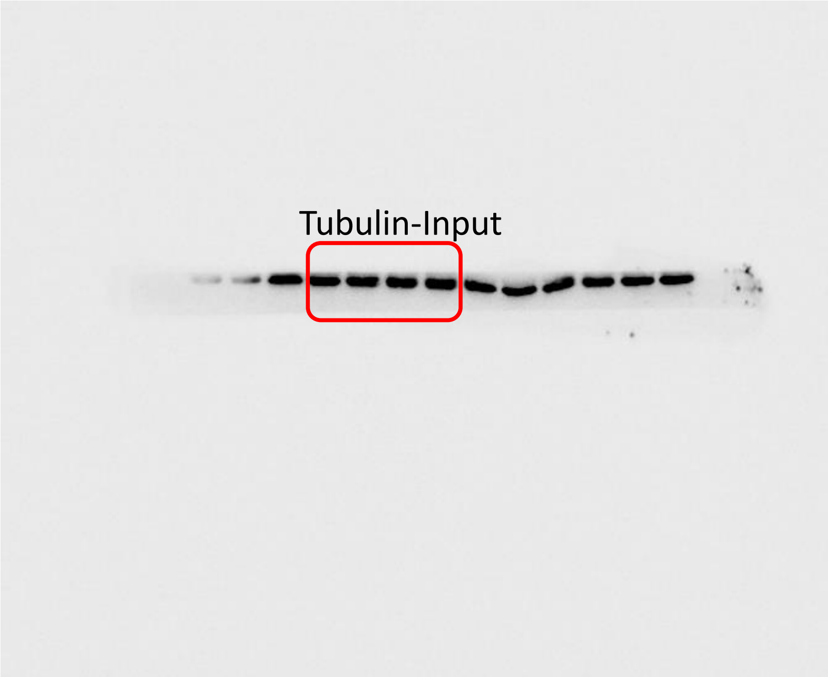

Figure 6F

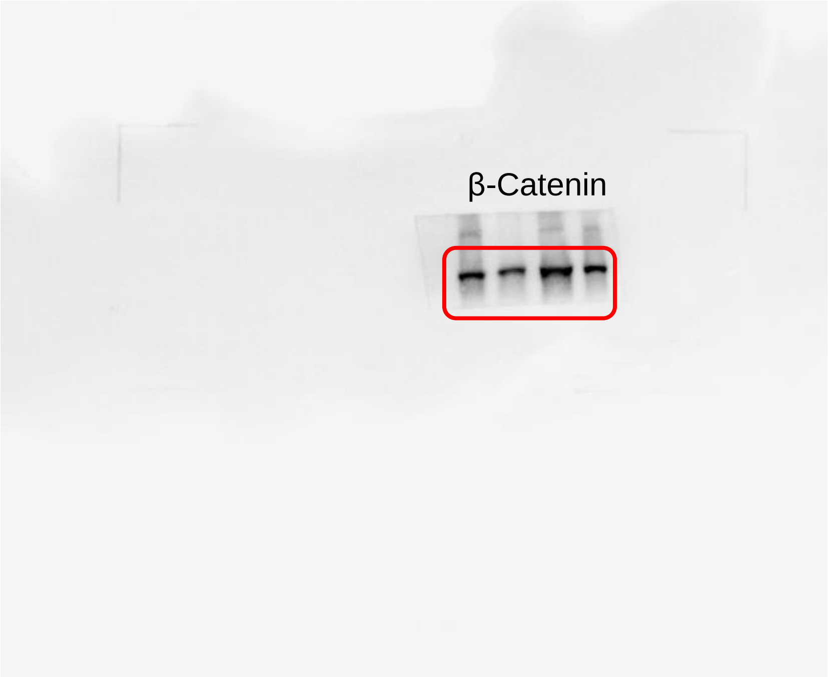

Figure 6F

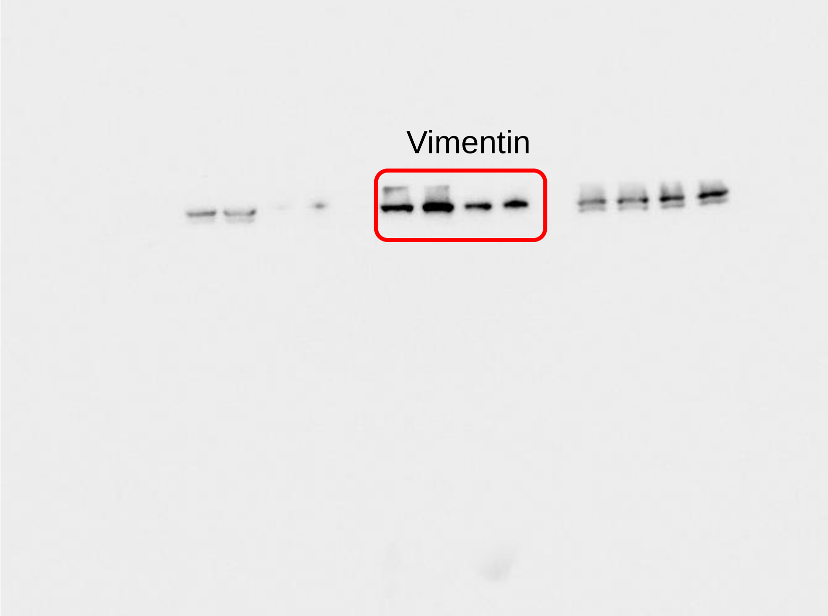

Figure 6F

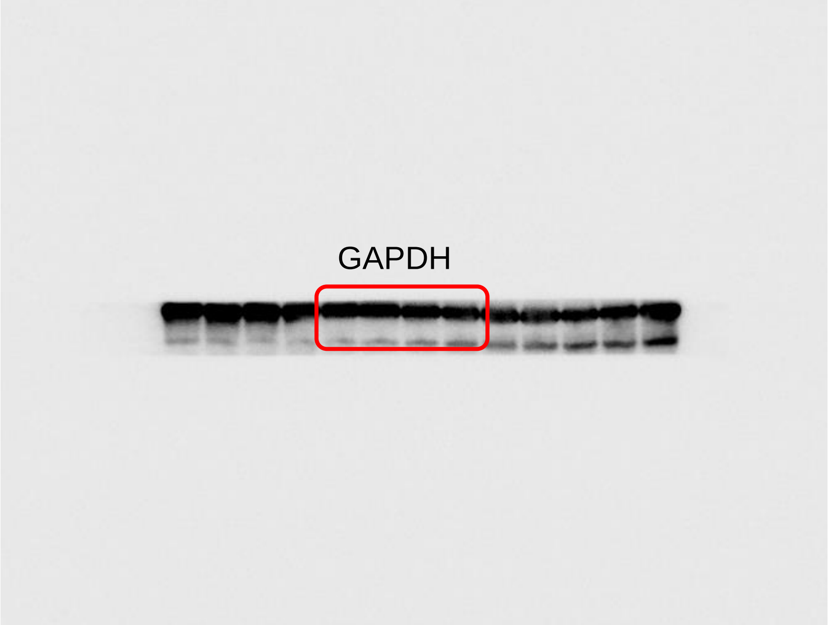

Supplement: Supplementary file 5 — Additional file 4. Uncropped images of western blot analysis. [file 12964_2023_1060_MOESM5_ESM.pdf]
